# Analysis of *CNGC* Family Members in *Citrus clementina* (Hort. ex Tan.) by a Genome-Wide Approach

**DOI:** 10.3390/ijms26030960

**Published:** 2025-01-23

**Authors:** Yuanda Lv, Shumei Liu, Yanyan Ma, Lina Hu, Huaxue Yan

**Affiliations:** 1 Institute of Fruit Tree Research, Guangdong Academy of Agricultural Sciences, Guangzhou 510640, China; lvyuanda2008@163.com (Y.L.);; 2 Key Laboratory of South Subtropical Fruit Biology and Genetic Resource Utilization, Ministry of Agriculture and Rural Affairs, Guangzhou 510640, China; 3 Guangdong Provincial Key Laboratory of Science and Technology Research on Fruit Tree, Guangzhou 510640, China

**Keywords:** *CNGC*, citrus, phylogenetic tree, promoter, *Phytophthora nicotianae*

## Abstract

The study focuses on the Cyclic nucleotide-gated ion channels (CNGCs) proteins in citrus, aiming to investigate their potential roles. A total of 33 CcCNGC proteins were identified and characterized in *Citrus clementina* using a genome-wide method. The study revealed that these proteins share a conserved CNGC domain structurally but exhibit significant differences in their primary sequence and motif composition. Phylogenetic analysis classified the CcCNGC proteins into 13 subgroups. The cis-elements present in all *CcCNGCs* promoters were identified and classified, and the number of elements was determined. The results suggested that these genes play important roles in citrus growth and development, as well as in response to biotic and abiotic stresses. Gene expression analysis further supported these findings, demonstrating that *CNGC* genes were responsive to various plant hormones and *Phytophthora nicotianae* infection, which causes citrus foot rot. Overall, the study indicated that members of the *CcCNGC* gene family exhibit structural and functional diversity. Further research is needed to validate the specific functions of individual family members and their roles in citrus physiology and response to stress conditions.

## 1. Introduction

It has been reported that calcium ions (Ca^2+^) play a central and crucial role as a second messenger in all eukaryotes, participating in a wide range of biological processes [1]. In plants, Ca^2+^ is vital in various physiological, biochemical, and metabolic processes. It is involved in regulating plant growth, development, and biological functions and in responding to both biotic and abiotic stresses, as well as in the immunity triggered by pathogen-associated molecular patterns (PAMPs) [2,3]. Upon detecting stress, plants rapidly mobilize Ca^2+^ as a specific signal. Several Ca^2+^ channels have been identified in plants, such as cyclic nucleotide-gated channels (CNGCs), glutamate receptor-like proteins (GLRs), reduced hyperosmolality-induced [Ca^2+^]_cyt_ increase channels (OSCAs), two-pore channels (TPCs), and others [4,5,6]. Among these channels, members of the *CNGC* family have been demonstrated to be crucial in plant development and stress resistance.

Cyclic nucleotide-gated ion channels (*CNGCs*) are a family of evolutionarily conserved genes found in animals, plants, and some prokaryotes, playing essential biological roles in vivo. Plant *CNGCs* were initially identified in barley [7,8]. The core structure of the CNGC protein comprises six transmembrane domains (S1–S6) [9], with the S4 transmembrane region serving as a positively charged voltage receptor [10]. A porous structure known as the P-ring is located between S5 and S6, containing an ion conduction pore region of 20 to 30 amino acids that is crucial for ion selectivity and serves as a distinguishing feature of CNGC proteins [11]. The cyclic nucleotide-binding domain (CNBD) and calmodulin-binding domain (CaMBD) in plant CNGC protein are both situated at the C-terminal, with the two domains partially overlapping [12,13]. In contrast, the CaMBD domain of animal CNGC protein is located at the N-terminal, while the CNBD domain is positioned at the C-terminal, providing a distinctive characteristic of animal CNGC proteins compared to plant CNGC proteins [13]. It has been observed that the binding of cyclic nucleotides to the CNBD results in the regulation of protein conformation, leading to the activation of CNGCs, the opening of ion channels, and the inflow of extracellular Ca^2+^. To avoid excessive intracellular Ca^2+^ levels, calmodulin (CaM) binds to the CaMBD domain, preventing cNMPs from binding to CNBD and, subsequently, closing channel gating [14]. Moreover, studies have shown that CaM can also bind to isoleucine glutamine (IQ) motifs, adding complexity to the ligand regulation of plant *CNGCs* [15,16]. *CNGCs* have been identified in numerous plant species, including dicots and monocots [17,18].

Previous studies have demonstrated the significant role of *CNGCs* in regulating various physiological processes in plants, such as growth and development, cell death, immune response, and responses to both biotic and abiotic stress. Specifically, *CNGCs* play a crucial role in plant growth and development, particularly in the growth and development of pollen tubes. Studies have shown that the expression of *AtCNGC7*, *AtCNGC8*, *AtCNGC16*, and *AtCNGC18* is limited to pollen development and the gametophyte [19]. Furthermore, the function of *AtCNGC18* in *Arabidopsis thaliana* has been confirmed to be specifically expressed in pollen grains [9,16]. In *Pyrus bretschneideri*, the *PbrCNGC14-18*, *PbrCNGC2*, *PbrCNGC7-9*, and *PbrCNGC12-13* are specifically expressed in pollen [20]. In Arabidopsis, proteins CNGC18, CNGC7, and CNGC8 are involved in pollen tube growth, with their functions being redundant; additionally, CNGC14, CNGC5, CNGC6, and CNGC9 are essential for regulating root hair growth [21]. In rice, *CNGC13*, which is homologous to *AtCNGC19*, has been shown to play a similar role in pollen tube growth [22,23].

The *CNGCs* play a crucial role in responding to abiotic stress. In Arabidopsis, *AtCNGC10* was shown to negatively regulate salt stress [9,24,25], while both *AtCNGC19* and *AtCNGC20* genes were found to be involved in responding to salt stress [26]. In *Amaranthus hypochondriacus*, the expression of *AhCNGC5* and *AhCNGC17* significantly changed under salt stress conditions, aligning with the roles of *AtCNGC5* and *AtCNGC17* in *A. thaliana* [27,28,29]. In *Nicotiana tabacum*, *NtabCNGC6* and *NtabCNGC7* are involved in Cd stress and cold stress, with an upregulation of expression [25,30]. In Arabidopsis, CNGCs were found to be involved in the uptake and transport of Pb^2+^ or Cd^2+^ ions, with *AtCNGC1*, *AtCNGC10*, *AtCNGC13*, and *AtCNGC19* functioning in Pb^2+^ toxicity, and *AtCNGC11*, *AtCNGC13*, *AtCNGC16*, and *AtCNGC20* playing roles in Cd^2+^ toxicity [31]. In *N. tabacum*, NtabCNGCs were found to be involved in drought stress in the late stage [30]. As reported, *AtCNGC16* was found to be involved in pollen fertility under heat and drought stress [9,32]. In Arabidopsis and moss, *AtCNGC2* and *CNGCb* have been identified as regulators of the heat shock response (HSR) and acquired thermotolerance in plants [30]. Additionally, *AtCNGC6* has also been shown to play a role in HSR. In rice, *OsCNGC14* and *OsCNGC16* have been found to regulate cytosolic Ca^2+^ signals in response to temperature stress [30,33]. In the *Brassica oleracea* genome, there are 26 *CNGC* genes, with 13 *BoCNGC* genes identified as responding to cold stress [34]. In *Mangifera indica* fruit peel, after two days of cold stress, there was an upregulated expression of *MiCNGC15*, indicating a role for *CNGC* in responding to decreases in temperature [35].

*CNGCs* regulate Ca^2+^ influx and the resulting Ca^2+^ signaling. Recent research has shown that CNGC proteins are involved in plant immunity, particularly in the HR triggered by pathogens. In Arabidopsis, certain *CNGCs* have been identified as key regulators of SA or resistance gene (*R* gene) mediating responses to pathogen infections [36,37,38,39], such as *AtCNGC2* [14], *AtCNGC4* [40], *AtCNGC11*, and *AtCNGC12* [38,41]. Studies have demonstrated that mutants of *AtCNGC2* and *AtCNGC4* (*dnd1* and *dnd2*) exhibit enhanced broad-spectrum resistance to bacterial pathogens and are essential for effector-triggered immunity (ETI) via Ca^2+^ signaling [36,40,42]. In potato and tomato, downregulation of the *AtCNGC2* ortholog has been linked to increased resistance to late blight and powdery mildew. However, this resulted in dwarfed and necrosis in tomato, while no such effects were observed in potato [43]. These results emphasize the unique interactions between *AtCNGC2* homologous genes and pathogens in various plant species. Furthermore, *AtCNGC2* is known to play a role in DAMP perception, as well as in the production of reactive oxygen species (ROS) and/or nitric oxide (NO), and the activation of ET/JA pathways in the absence of SA signaling [39,44,45]. The double mutation of *AtCNGC11* and *AtCNC12* (*cpr22*) has been shown to increase resistance to *Peronospora parasitica* Emco5 [38]. In rice, *CNGCs* have been demonstrated to play a significant role in stress resistance [46]. Additionally, *AtCNGC19* has been shown to be important for defense against the fungal pathogen *Botrytis cinerea* [23,47]. In the apple, gene editing of the homolog of *AtCNGC2*, named *MdCNGC2* in apple “orin” callus, was conducted. The results showed that editing the *MdCNGC2* gene enhanced immune responses and improved resistance to *Botryosphaeria dothidea* in apple callus [7]. Analysis of the *CNGC* family in apple (*Malus domestica*) and pear (*Pyrus bretschneideri* and *Pyrus communis*) in response to threats of Valsa canker revealed the presence of many cis-acting regulatory elements responsive to various stresses and hormones in the promoters of *CNGCs*. Subsequent overexpression of *MdCN11* and *MdCN19* in apple fruits and ‘Duli’ (*Pyrus betulifolia*) suspension cells resulted in decreased resistance to Valsa canker. This suggested that *MdCN11* and *MdCN19* play a negative role in response to Valsa canker by inducing HR [39]. Together, these studies demonstrated the importance of *CNGCs* in plant disease resistance.

As a non-selective cation channel essential for regulating plant growth, development, and stress resistance, the role of *CNGCs* in response to abiotic stress remains largely unexplored in citrus. In this study, bioinformatics tools were employed to comprehensively analyze the citrus *CNGC* gene family, examining gene structure, protein-conserved domains, cis-acting elements within promoter segments, and their expression profiles under different abiotic stress conditions. The findings aim to provide valuable insights for future research on the role of *CNGCs* in citrus.

## 2. Results

### 2.1. Identification of CcCNGC Gene Family Members

Using the protein sequences of CNGC family members in *A. thaliana* as a reference, potential *CNGC* members in citrus were identified through bidirectional homology blastp comparison. Further screening based on conserved domains resulted in the identification of a total of 33 *CNGC* genes in the *C. clementina* genome, temporarily named *CcCNGC1-33*. To better understand the structure and function of *C. clementina CcCNGC* genes, the sequence features of citrus *CcCNGCs* were analyzed using the Expasy website. The physicochemical properties of the 33 CcCNGC proteins, including gene ID, amino acid number, molecular weight, isoelectric point, and total average hydrophobicity, were also analyzed. The length of these *CcCNGC* genes ranged from 1335 to 2664 amino acids (Table 1), with an average length of approximately 2038 amino acids. The amino acid number of CcCNGC protein was between 445 and 888, with an average of 680. The theoretical isoelectric point was between 6.42 and 9.51. The instability index ranged from 35.55 to 48.11, with only CcCNGC1, CcCNGC15, CcCNGC23, CcCNGC27, CcCNGC30, and CcCNGC31 proteins having an instability index of less than 40, while the remaining 27 CcCNGC proteins had an instability index greater than 40, indicating that these proteins are unstable. The aliphatic index was between 24.51 and 32.06, indicating an average level of thermal stability. The average hydropathy score was between 0.616 and 0.805, which means that all CcCNGCs are hydrophobic proteins. Subcellular localization prediction of citrus CcCNGC proteins indicated that, with the exception of CcCNGC15, which lacks a predicted localization, all other CcCNGCs are localized on the plasma membrane.

### 2.2. Phylogenetic Analysis of the CNGC Gene Family

**Figure 1:**
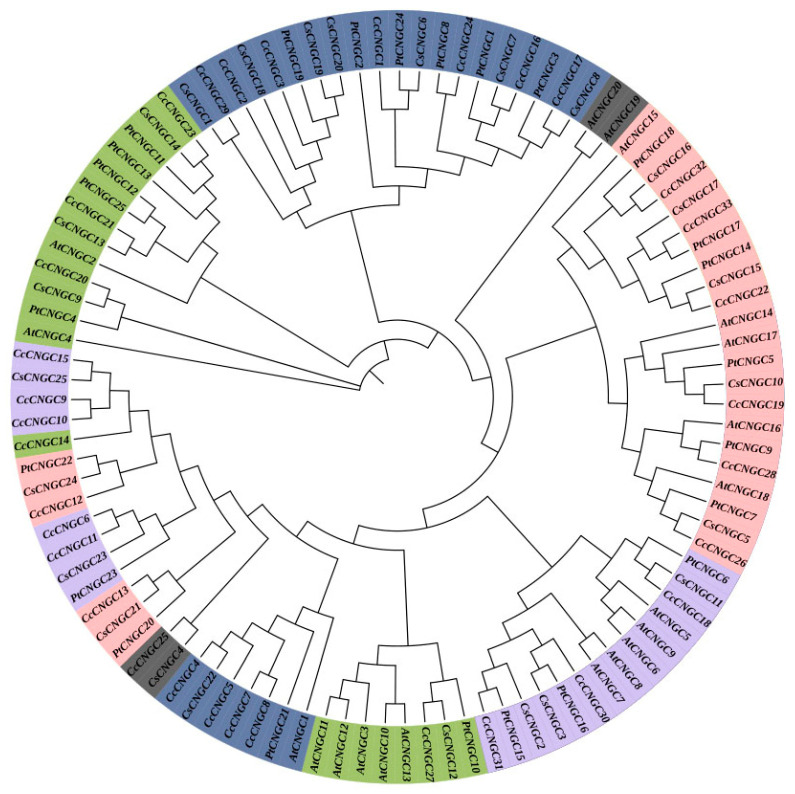
Phylogenetic tree of citrus CNGC protein and Arabidopsis CNGC Protein. Protein name: *C. clementina* CcCNGC proteins, *C. sinensis* CsCNGC proteins, *P. trifoliata* PtCNGCs, *A. thaliana* AtCNGCs. Different color blocks represent different subgroups.

To understand the evolutionary relationship between the *CNGC* gene family in citrus and model plants, a phylogenetic tree was constructed between citrus *C. clementina* (33), *C. sinensis* (25), *P. trifoliata* (25), and *A. thaliana* (20) *CNGC* gene family members using conservative amino acid sequences with MEGA 6.0 software. The *CNGC* members in Arabidopsis and citrus were divided into 13 subgroups, with the largest subgroup having 22 members and the smallest subgroup having only 1 (Figure 1). Many of these subgroups contained *CNGC* genes from *C. clementina*, *C. sinensis*, *P. trifoliata*, and *Arabidopsis*. In the analysis of the 33 *CNGC* genes in *C. clementina*, *CcCNGC20* was identified as a separate branch, suggesting a possibly lower homology with other members. The results of the phylogenetic analysis indicated a potential correlation between the subfamily classification of citrus *CNGC* genes and their functional similarity.

### 2.3. Gene Structure, Conserved Motifs, and Domain Analysis of the CcCNGC Gene Family Members

**Figure 2:**
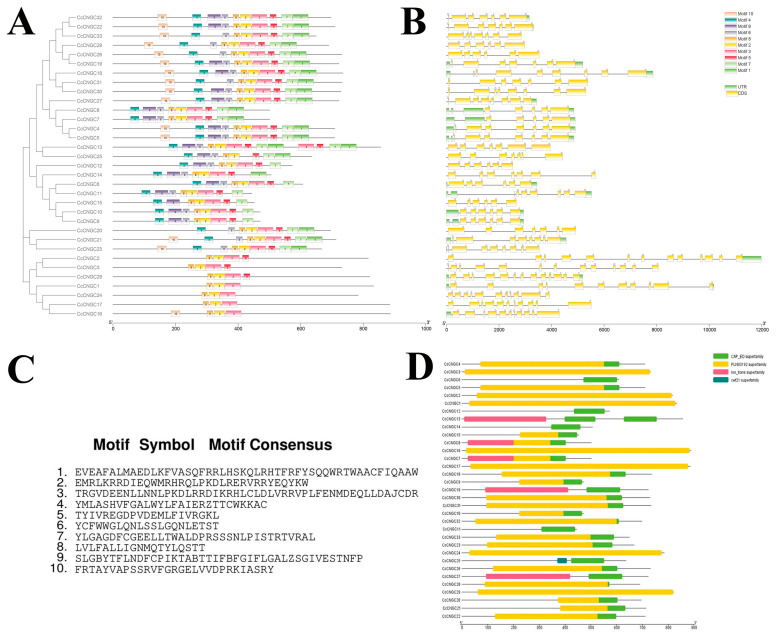
Phylogenetic relationship, gene structure analysis, conserved domain, and conserved motifs analysis of *CNGC* gene family of *C. clementina*. (**A**) Conserved motifs analysis of *CcCNGCs*; (**B**) Gene structure analysis of *CcCNGCs*; (**C**) The motif symbol; (**D**) Conserved domain of *CcCNGCs*.

To obtain the gene structure and conserved domains of *CNGC*, analysis was performed on 33 *CcCNGC* members. By aligning the CDS of *CcCNGC* to the corresponding genome sequences, the distribution of introns, exons, and UTRs of these 33 genes was analyzed. The number of introns in citrus *CcCNGC* gene family members ranged from 4 to 12, while the number of exons ranged from 5 to 13. Among them, *CcCNGC2*, *CcCNGC3*, and *CcCNGC29* had the highest number of CDS, and some members did not have UTR, possibly due to incomplete genome annotation. An interesting observation was that members with close phylogenetic relationships show greater similarity in gene structure, as shown in Figure 2. Conserved motif analysis using the online software MEME revealed 10 motifs in the *CcCNGC* gene family, with similar motifs and arrangement order among most genes within the family. Motif 2, motif 3, and motif 8 were present in all *CcCNGC* genes, indicating that these motifs are characteristic conserved motif sequences in the evolution of *CcCNGCs*. Predictions of conserved domains in *CNGC* family members showed that all CNGC members contain the CAP-ED superfamily conserved domain, and most family members contain the PLN03192 superfamily conserved domain (Figure 2).

### 2.4. Chromosomal Localization and Synteny Analysis of CNGC Gene Family Members

**Figure 3:**
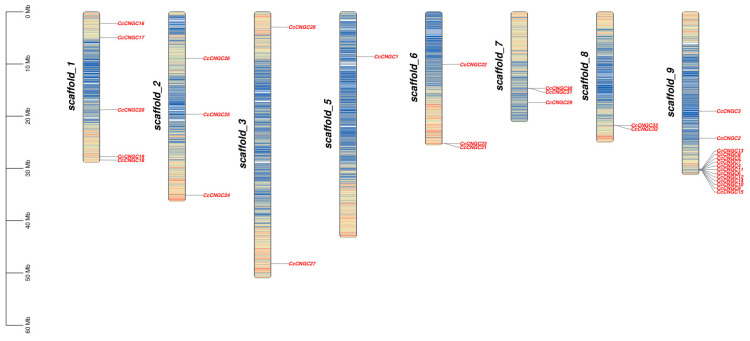
Location analysis of *C. clementina CNGC* gene family on chromosome.

**Figure 4:**
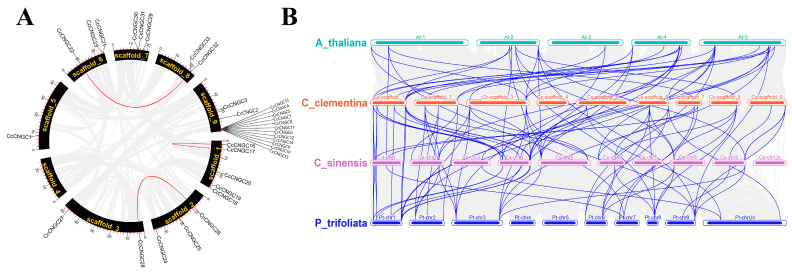
Colinearity analyses of *CNGC* genes. (**A**) Colinearity analyses in *C. clementina*; (**B**) Colinearity analyses in *C. clementina*, *C. sinensis*, *P. trifoliata*, and *A. thaliana*.

The results revealed that, with the exception of chromosome 4, genes were spread across the remaining 8 chromosomes, displaying an uneven distribution among them (Figure 3). Among the members of the *CcCNGC* gene family, chromosome 9 had the highest abundance, with 15 members. Conversely, chromosome 5 had the lowest number of members, with only one, while chromosome 4 did not contain any *CNGC* gene family members. This indicated that the distribution of *CNGC* gene family members on each chromosome is not correlated with the chromosome’s length. We conducted intra-species synteny analysis of the *CcCNGC* gene family, which showed that *CcCNGC22* was in the same syntenic region as *CcCNGC33* and *CcCNGC32*; *CcCNGC16* was in the same syntenic region as *CcCNGC17*; and *CcCNGC28* was in the same syntenic region as *CcCNGC26* (Figure 4). Furthermore, inter-species synteny analysis was performed on *C. clementina*, *C. sinensis*, *P. trifoliata*, and *A. thaliana CNGC* gene families. The results showed that there were more synteny modules among several species, with more synteny between *C. clementina*, *C. sinensis*, and *A. thaliana* compared to *P. trifoliata*.

### 2.5. Analysis of Cis-Acting Elements in the Promoter Regions of the CcCNGCs

**Figure 5:**
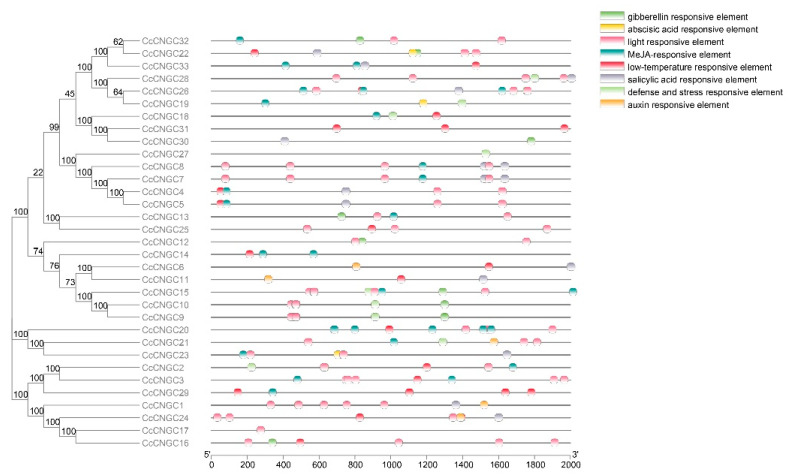
Cis-acting element distribution in *CcCNGCs* promoters.

To further understand the potential regulatory mechanism of *CNGCs* in *C. clementina*, we conducted cis-acting element analysis on the 2000 bp upstream promoter region. The predicted results revealed that out of 33 *CcCNGC* promoters, 8 elements were identified (Figure 5), including abscisic acid response elements (ABRE), methyl jasmonate response elements (TGACG-motif), auxin response elements (TGA-element), salicylic acid response elements (TCA), and gibberellin response elements. The promoter region also included elements related to biological rhythms control, such as light response elements, low-temperature response elements, as well as defense and stress response elements. The specific number of elements is shown in Supplemental Figure S1. There were different types and quantities of cis-acting elements among *CcCNGC* members, indicating that *CcCNGC* members have different biological functions and are closely related to hormone signal transduction pathways and stress responses.

### 2.6. Expression Analysis of the CcCNGCs in Citrus Tissues

**Figure 6:**
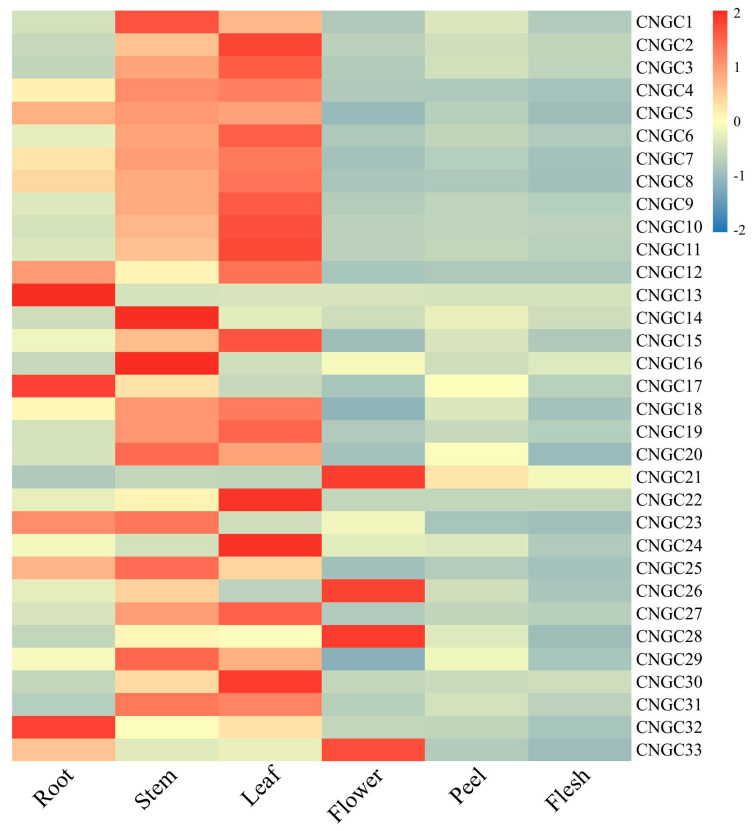
The expression level of *CcCNGC* genes in different parts of *C. clementina*.

The expression of *CNGC* gene family members in different tissues of *clementina*, including roots, stems, leaves, flowers, peel, and pulp, was detected. It was observed that the expression levels of *CNGC* family members in the peel and flesh were significantly lower compared to other tissues, while the stems and flowers showed lower expression levels compared to roots and leaves. Thirteen members, including *CcCNGC5*, *CcCNGC13*, *CcCNGC25,* and *CcCNGC32*, exhibited the highest expression in roots (Figure 6). Four members, including *CcCNGC14* and *CcCNGC16*, had the highest expression in stems. Over 20 members, including *CcCNGC2* and *CcCNGC5*, exhibited relatively high expression in leaves. Five members, including *CcCNGC10*, showed relatively high expression in leaves. *CcCNGC16* and *CcCNGC21* exhibited higher expression in the flesh compared to other members.

### 2.7. Expression of CcCNGCs Under Plant Hormone Treatment

**Figure 7:**
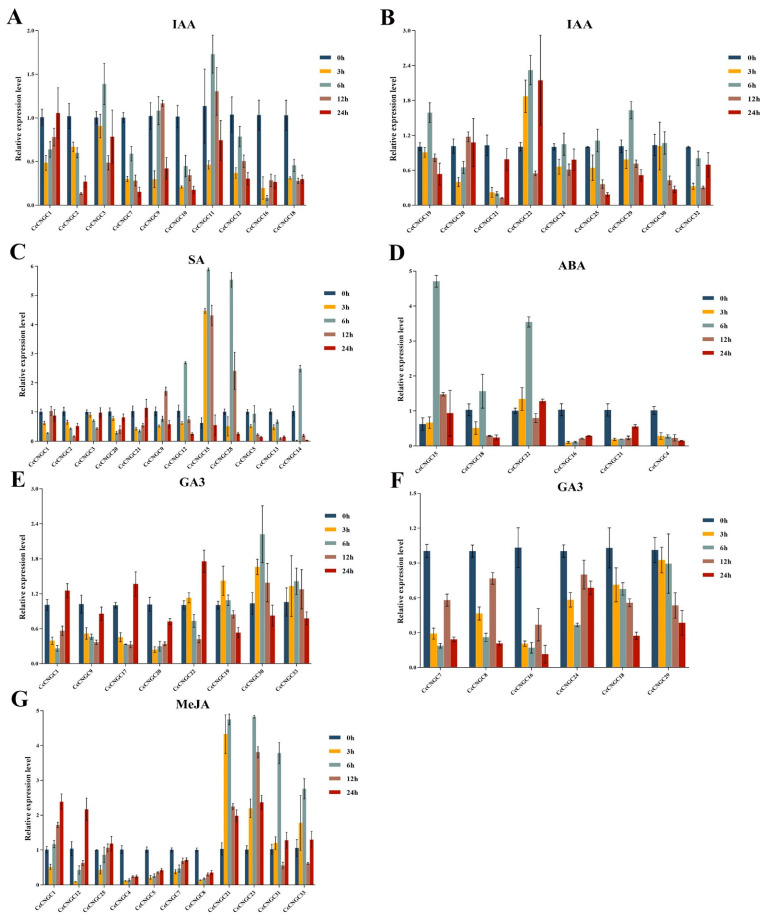
The expression level of *CcCNGC* genes under plant hormone treatment. (**A**,**B**) The expression level of *CcCNGC* genes under IAA treatment; (**C**) The expression level of *CcCNGC* genes under SA treatment; (**D**) The expression level of *CcCNGC* genes under ABA treatment; (**E**,**F**) The expression level of *CcCNGC* genes under GA3 treatment; (**G**) The expression level of *CcCNGC* genes under MeJA treatment.

The expression of *CcCNGCs* was found to be modulated by plant hormone treatments (Figure 7 and Supplemental Figure S2). Results indicated that *CcCNGC15*, *CcCNGC17*, *CcCNGC23*, *CcCNGC26*, *CcCNGC28*, *CcCNGC31*, and *CcCNGC33* were significantly upregulated under IAA treatment. *CcCNGC15* demonstrated *5* with a nearly 5-fold upregulation. *CcCNGC4*, *CcCNGC5*, *CcCNGC6*, *CcCNGC8*, *CcCNGC13*, *CcCNGC14*, and *CcCNGC27* were significantly downregulated by IAA induction, with *CcCNGC27* showing the highest downregulation. Under SA treatment, the expression of *CcCNGC1*, *CcCNGC2*, *CcCNGC3*, *CcCNGC20*, and *CcCNGC21* followed an inverted V-shape, while *CcCNGC9*, *CcCNGC12*, *CcCNGC15*, and *CcCNGC28* followed a parabolic pattern, and *CcCNGC5*, *CcCNGC13*, and *CcCNGC14* were significantly downregulated at 3 h. Under GA3 treatment, the expression of *CcCNGC1*, *CcCNGC9*, *CcCNGC17*, *CcCNGC20*, and *CcCNGC21* followed an inverted V-shape, while *CcCNGC19*, *CcCNGC28*, *CcCNGC30*, and *CcCNGC33* followed a parabolic pattern. *CcCNGC7*, *CcCNGC8*, *CcCNGC16*, and *CcCNGC24* showed a decrease in expression at 3 h and 6 h after treatment but an increase at 12 h; however, the expression levels were always lower than 0 h. Notably, under GA3 treatment, *CcCNGC18* and *CcCNGC29* always showed downregulation. Under ABA treatment, the expression of *CcCNGC15*, *CcCNGC18*, and *CcCNGC22* showed a parabolic pattern, with the expression of *CcCNGC16* and *CcCNGC21* decreasing at the beginning of treatment, then increasing continuously but lower than 0 h. The gene *CcCNGC4* was consistently downregulated. Under MeJA treatment, the expression of *CcCNGC1*, *CcCNGC12*, and *CcCNGC25* significantly decreased at 3 h, then gradually increased at 6 h, 12 h, and 24 h, with expression levels at 24 h higher than at 0 h. The expression of *CcCNGC4*, *CcCNGC5*, *CcCNGC7*, and *CcCNGC8* gradually increased at 6 h, 12 h, and 24 h, but the expression levels at 24 h were significantly lower than at 0 h. The expression levels of *CcCNGC21*, *CcCNGC23*, *CcCNGC31*, and *CcCNGC33* exhibited a parabolic pattern, with peak levels observed at 6 h.

### 2.8. Expression of CcCNGCs Under Low-Temperature and Light Stress

**Figure 8:**
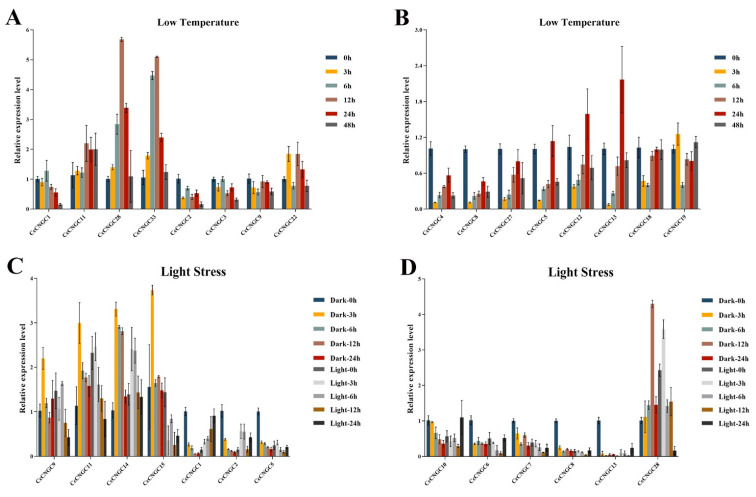
The expression level of *CcCNGC* genes under low-temperature and light stress. (**A**,**B**) The expression level of *CcCNGC* genes under low-temperature treatment; (**C**,**D**) The expression level of *CcCNGC* genes under light stress.

Under low-temperature stress, the expression of *CcCNGC* showed varying degrees of change. The expression of *CcCNGC1*, *CcCNGC11*, *CcCNGC28*, and *CcCNGC33* followed a parabolic pattern, but the vertex of the parabola occurred at different time points such as 6 h, 12 h, or 24 h. Some members of the *CcCNGC* family, such as *CcCNGC2*, *CcCNGC3*, *CcCNGC9*, and *CcCNGC22*, displayed a wave-like expression pattern. Additionally, there were some members whose expression decreased continuously at 3 h, 6 h, and 12 h but increased at 24 h. In this type of variation, the expression of *CcCNGC4*, *CcCNGC8*, and *CcCNGC27* at 24 h was significantly lower than at 0 h, while the expression of *CcCNGC5*, *CcCNGC12*, and *CcCNGC13* was noticeably lower than at 0 h. After 48 h of low-temperature stress treatment, except for *CcCNGC11*, *CcCNGC18*, and *CcCNGC19*, the expression of the rest of the *CNGC* family members decreased (Figure 8 and Supplemental Figure S3).

The expression levels of *CNGC* family members show distinct responses when exposed to dark or light treatments (Figure 8 and Supplemental Figure S3). Apart from *CcCNGC9*, *CcCNGC11*, *CcCNGC14*, and *CcCNGC15*, all other *CNGC* members showed upregulation in expression after 3 h of dark treatment. *CcCNGC1* exhibited a perfect parabolic shape, with expression decreasing gradually under dark treatment and gradually recovering to the 0-h level after light exposure. *CcCNGC2*, *CcCNGC5*, and *CcCNGC10* showed a gradual decrease in expression under dark treatment and a sudden increase after light exposure, but with less regularity after light exposure. *CcCNGC6*, *CcCNGC7*, and *CcCNGC8* showed more regular changes after light exposure. *CcCNGC13*, *CcCNGC14*, and *CcCNGC28* displayed significant changes in expression after dark treatment and recovery from light treatment. Notably, *CcCNGC28* showed a 5-fold increase in expression at 0 h after recovery from light treatment.

### 2.9. Expression and Validation of CcCNGCs Under Phytophthora Treatment

**Figure 9:**
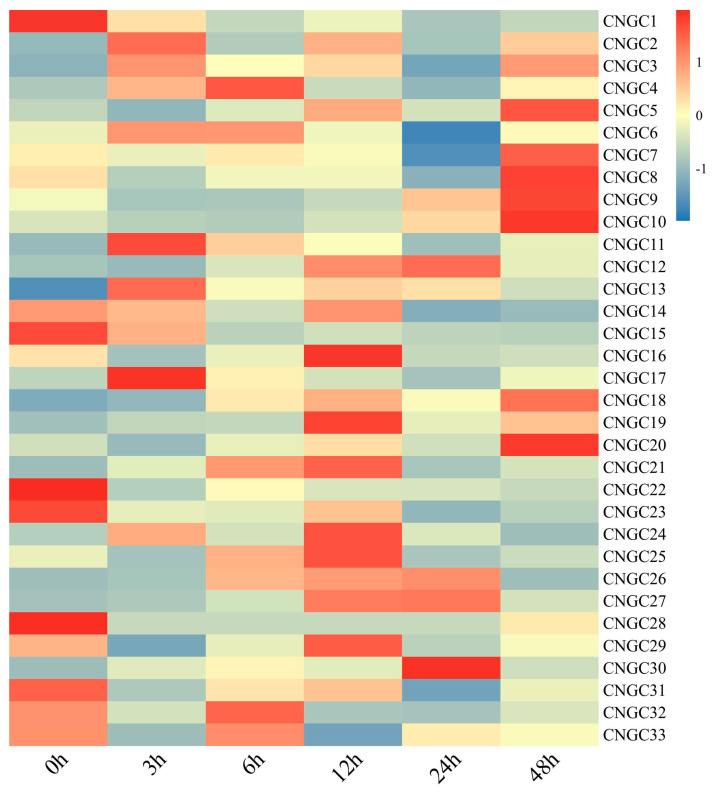
The expression level of *CcCNGCs* at different times after infection with *P. nicotianae* in *C. clementina*.

Changes in the expression levels of *CNGC* genes were shown induced by *P. nicotianae* within 48 h after being infected with the pathogen (Figure 9). Eight genes, including *CcCNGC9*, *CcCNGC10*, *CcCNGC12*, *CcCNGC20*, *CcCNGC21*, *CcCNGC24*, *CcCNGC27*, and *CcCNGC30*, were highly induced by *P. nicotianae*, with peak inductions exceeding 4-fold for *CNGC30* and 6-fold for *CNGC27*. Four distinct patterns were observed, namely gradual induction, early induction, late induction, and mid-term induction. Specifically, *CcCNGC3*, *CcCNGC5*, *CcCNGC27*, and *CcCNGC30* exhibited gradual induction, while *CcCNGC11* and *CcCNGC17* displayed early induction. The genes *CcCNGC9*, *CcCNGC10*, *CcCNGC12*, and *CcCNGC20* showed late induction with peak expression levels at 48 h, while *CcCNGC16*, *CcCNGC21*, and 24 peaked in mid-term induction. In contrast, *CcCNGC1*, *CcCNGC15*, and *CcCNGC28* were found to be moderately inhibited or not induced by *Phytophthora* infection (Figure 9).

**Figure 10:**
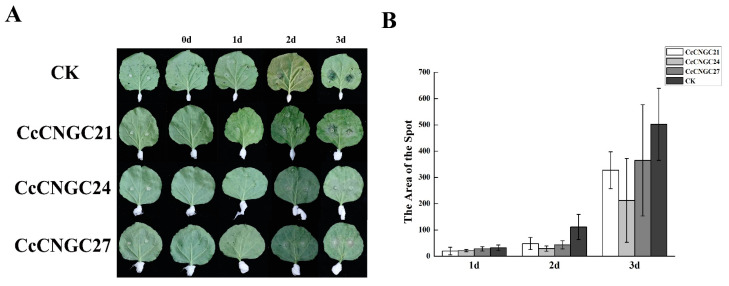
The symptoms of the tobacco leaves after infection with *P. nicotianae* in transient transformation *CcCNGC21*, *CcCNGC24*, and *CcCNGC27*. (**A**) Phenotypic changes of tobacco leaves; (**B**) The area of the spot used ImageJ.

Based on the quantitative results, *CcCNGC21*, *CcCNGC24*, and *CcCNGC27* exhibited significant responses to *Phytophthora* stress and then overexpressed in the tobacco leaves by the transient transformation. It was found that all infected leaves showed disease symptoms after tobacco plants were infected with *Phytophthora*, with a disease incidence rate of 100%. It was observed that the disease spots gradually expanded after inoculation. The disease spot on *CcCNGC27* showed the greatest change on day 3 post-inoculation, but the spot area was smaller than that of the control group. This indicated that overexpression of the *CcCNGC21*, *CcCNGC24*, and *CcCNGC27* genes negatively affected the disease development spots, which were smaller than in the control group (Figure 10).

## 3. Discussion

CNGC proteins are channels located on the plasma membrane and have been discovered in various plant species. Numerous functional studies have demonstrated their crucial role in regulating plant growth and development, as well as in defending against pathogens [9,48,49,50]. While the *CNGC* gene family has been extensively studied in various citrus and their closely related species, including *C. sinensis*, *Citrus reticulata*, *Citrus grandis*, *Atalantia buxifolia*, and *P. trifoliata*, particularly in relation to their response to drought stress as observed in a recent study by Komal Zia et al. (2022), their role in disease resistance within the Citrus genus remains relatively unexplored [48]. Therefore, the primary aim of this research is to conduct a thorough investigation into the *CNGC* gene family within *C. clementina* and explore its potential role in combating *P. nicotianae* infections and responses to hormone treatments.

According to bioinformatics analysis, 33 *CcCNGC* members were identified in *C. clementina*, 25 in *C. sinensis*, 25 in *P. trifoliata*, and 20 in *Arabidopsis*. This suggested that the CNGC gene family is conserved across different species, with some functional differences. Analysis of the evolutionary tree of *CNGC* families in various species revealed that CNGC proteins in citrus and certain members in Arabidopsis show high conservation. However, *AtCNGC19* and *AtCNGC20* formed separate evolutionary clades, indicating species-specific differences. The grouping of other *CNGC* family members suggested structural conservation but varying functions across species.

The gene structure of *CcCNGCs* includes exons and introns. Xu suggested that the introns and exons evolved through acquisition, loss, insertion, or deletion. Gene structure plays a crucial role in determining gene function and serves as a fundamental basis for understanding the evolution of gene families [51]. This study revealed that the intron numbers of *CcCNGCs* vary from 4 to 12, while the exon numbers range from 5 to 13. It was hypothesized that the structure and function of *CcCNGCs* undergo changes as different introns/exons are inserted or deleted during the evolutionary process. Most genes in *CcCNGCs* possess 6–8 exons, indicating conservation within this gene family. Guo proposed that changes in intron/exon numbers during genome evolution could lead to chromosome fusion or recombination in organisms, thereby altering gene function [52]. In this study, the number of introns or exons in *CcCNGCs* genes was found to impact their biological function in *C. clementina*. A total of 10 motifs were identified in CcCNGC proteins, with motif 2, motif 3, and motif 8 primarily associated with encoding the CcCNGC domain. These motifs vary among different CcCNGCs, enhancing the functional diversity of CcCNGC proteins. Additionally, specific conserved motifs play a crucial role in determining their functions.

Based on the chromosome location analysis, it was found that 33 *CcCNGC* genes were unevenly distributed on 8 chromosomes in *C. clementina*, with the exception of chromosome 4. This indicates that *CcCNGC* genes are widespread in *C. clementina*. There were 3 members each on chromosomes 2, 6, and 7, while chromosomes 3 and 8 each had two *CcCNGCs*. Further exploration is needed to understand the distribution of genes on these chromosomes. By conducting collinearity analysis of the *CcCNGC* gene family among different species, the evolutionary relationship of the *CcCNGCs* can be better understood. The results of the collinearity analysis revealed that *CcCNGC22*, *CcCNGC33*, and *CcCNGC32*; *CcCNGC16* and *CcCNGC17*; as well as *CcCNGC28* and *CcCNGC26* were located in the same collinear regions, suggesting that these genes may have arisen from large-scale gene duplication during evolution. This indicates that the *CNGCs* in *C. clementina* underwent amplification during genome evolution. The collinearity analysis provides a theoretical basis for understanding the shared gene control loci between different species. Further collinearity analysis of *CNGC* genes among the *C. clementina*, *C. sinensis*, *P. trifoliata*, and *A. thaliana* genome revealed that *CNGC* genes exhibiting higher collinearity modules between *C. clementina* and the other two species are more closely related. Conversely, these displayed lower collinearity with *P. trifoliata*.

Based on our investigation of the tissue-specific expression patterns of the *CcCNGC* gene in *C. clementina*, we observed that *CcCNGCs* showed higher expression levels in stems and leaves compared to fruit peel and flesh. This finding is consistent with the analysis of elements in the gene promoters, which revealed a large number of light-responsive elements. This suggests that *CcCNGC* may be involved in photosynthesis in green tissues. Therefore, it is likely that *CcCNGCs* play a key role in green tissues and contribute to the process of photosynthesis. The functions of other elements in the promoter were found to be unrelated to fruit tissues. Our tissue-specific expression analysis indicated that the expression levels of the promoter were notably low in fruit peel and flesh. Only a select few members of the *CcCNGCs* family, such as *CcCNGC21*, *CcCNGC26*, *CcCNGC28*, and *CcCNGC33*, exhibited high expression levels in flowers, while genes like *CcCNGC13*, *CcCNGC16*, and *CcCNGC32* showed high expression levels in roots. However, the majority of *CcCNGCs* genes showed lower expression levels in roots and flowers, similar to what was observed in fruit peel and flesh.

The promoters may influence the function of the genes, so in this study, we analyzed the elements in the promoters. The results showed that *CcCNGCs* could play a role in the plant defense process. Firstly, the proteins were predicted to be localized in the plasma membrane, with main functions including control functions, energy conversion, material transport, information recognition, and transmission. These functions suggest that they may be involved in plant responses to stress. Secondly, the elements enriched in the promoters of the *CcCNGC* family were found to be similar and simple. It was observed that only defense and stress response elements, as well as some hormone response elements, exist in their promoters. This indicates that they are involved in stress regulation, particularly in osmotic stress regulated by ABA, salicylic acid, gibberellin, IAA, and MeJA. Additionally, light response and low-temperature response elements were also identified. Thirdly, *CcCNGC* expression can be induced by stress-related hormones and treatment with *P. nicotianae*.

The quantitative heat map reveals that the majority of elements in the *CcCNGC* promoters are associated with ABA, low temperature, light, and MeJA responses, indicating that the functions of *CcCNGC* are closely linked to these factors. ABA has a strong relationship with the *CNGC* family, as it has been shown that ABA-induced stomatal closure is crucial for cytoplasmic Ca^2+^ signaling in most plant stomata [53]. Studies have demonstrated that the Arabidopsis *CNGC* quadruple mutant *cngc5-1 cngc6-2 cngc9-1 cngc12-1* (c5/6/9/12) displays a significant ABA-insensitive stomatal closure phenotype [54]. In this study, *CcCNGC15* contains 7 ABA-responsive elements, with its expression levels significantly changing after ABA treatment. The expression at 6 h is more than four times higher than at 0 h. Other members of the *CcCNGC* family, such as *CcCNGC7*, *CcCNGC10*, *CcCNGC17*, *CcCNGC22*, and *CcCNGC28*, also show significant changes in expression levels and contain ABA-responsive elements. This aligns with the prediction that 24 members of the *CcCNGC* family have ABA-responsive elements, highlighting their active response to ABA hormone signaling. MeJA also influences leaf aging through calcium ions. The quantitative heat map shows that the promoter of *CcCNGC20* contains 12 MeJA response elements, while *CcCNGC26* contains 6 MeJA response elements, with expression changes observed after MeJA application. These findings suggest that the *CcCNGC* family plays a crucial role in plant hormone responses.

Similarly, low-temperature and light-responsive elements were found to be the predominant elements in the promoters of *CcCNGC* genes. The predicted results indicated that several members of the *CcCNGC* family, including *CcCNGC28*, *CcCNGC33*, and *CcCNGC13*, were particularly sensitive to low-temperature stress. Studies in *A. thaliana* have shown that *CNGC20* plays a positive role in regulating low-temperature stress by facilitating calcium influx [55]. Likewise, in rice, *OsCNGC14* and *OsCNGC16* have been shown to enhance Ca^2+^ influx under conditions of low temperature or heat stress [56]. Furthermore, the *CcCNGC* in *C. clementina* was also found to contain a significant number of light-responsive elements. This suggests that members of the *CcCNGC* family exhibit altered responses to light-induced stress. For instance, the expression of *CcCNGC9* and *CcCNGC15* was significantly altered in response to changes in light exposure, likely due to the presence of light-responsive elements. These findings are consistent with previous observations indicating that *CcCNGCs* are highly expressed in leaves.

As a result of the defense and stress-responsive elements in the promoter, we infected *clementina* with *P. nicotianae* and observed changes in the expression levels of almost all family members under pathogen stress. Upon transient transformation with overexpressed *CcCNGC21*, *CcCNGC24*, and *CcCNGC27*, the size of the lesions gradually increased but remained smaller than the control, indicating the response of these *CcCNGCs* to *P. nicotianae*. However, further verification and analysis are required to determine whether these genes confer disease resistance or susceptibility. While only a few family members showed this response in the heat map, it is clear that the *CNGC* family plays a role in plant immunity. In *A. thaliana*, *AtCNGC2* and *AtCNGC4* were well-studied members of the *CNGC* family, with mutant strains *dnd1* and *dnd2/hlm1* exhibiting defects in HR induction but still capable of ETI against non-toxic pathogens [57]. Another member, *AtCNGC6*, has been shown to mediate EATP-induced [Ca^2+^] cell signaling and contribute to plant immunity in response to *Pseudomonas syringae* mutants [58]. Additionally, in Arabidopsis *bak1/serk4* mutants, transcripts of *CNGC20* and *CNGC19* were elevated compared to wild-type plants, highlighting the important role of *CNGC20* and *CNGC19* in cell death regulation [59]. In rice, *OsCNGC9*, a homolog of Arabidopsis *CNGC18*, has been found to positively regulate resistance against rice blast [46].

## 4. Materials and Methods

### 4.1. Plant Materials

The materials used in this study are *C. clementina* (Hort. ex Tan.) from the greenhouse of the Fruit Research Institute of Guangdong Academy of Agricultural Sciences. Flowers, leaves, roots, peels, and flesh of *C. clementina* were collected in triplicate. These samples were rapidly frozen in liquid nitrogen and stored in a −80 °C freezer for further research.

### 4.2. Identification and Physicochemical Property Analysis of Citrus CNGC Genes

Genomic and gene annotation files of *C. clementina*, *Citrus sinensis*, and *Poncirus trifoliata* were downloaded from the citrus genome website (http://citrus.hzau.edu.cn/index.php, accessed on 15 March 2024), while the Arabidopsis CNGC protein sequences were obtained from the TAIR website (https://www.arabidopsis.org/). Using TBtools [60] for alignment, potential CNGC family genes in the citrus genome were screened out. The candidate genes were then submitted to NCBI-CD-Search (https://www.NCBI.nlm.nih.gov) and Pfam (http://Pfam.xfam.org/search, accessed on 16 March 2024) websites to validate if the selected genes contain the conserved domains of *CNGC* genes in *C. clementina*. After filtering and removing duplicate sequences, *CNGC* genes in the genomes of *C. clementina*, *C. sinensis*, and *P. trifoliata* were identified. Subsequently, the lengths, isoelectric points, hydrophobicity, and instability of the *clementina* CNGC proteins were analyzed using the ExPASy website (https://web.expasy.org/cgi-bin/protparam/protparam, accessed on 17 March 2024), and their subcellular localization was predicted using the Plant-mPLoc (http://www.csbio.sjtu.edu.cn) website.

### 4.3. Systematic Phylogenetic Characteristics of CNGC Family Members

To study the evolutionary relationship between the *CNGC* gene families of *C. clementina*, *C. sinensis*, *P. trifoliata*, and *Arabidopsis*, multiple sequence alignment analysis was conducted using DNAMAN software version 9.0, followed by manual correction. The MEGA software version 11.0 was then used to construct phylogenetic trees for these *CNGC* families and categorize them into respective groups.

### 4.4. Analysis of Conserved Motifs, Conserved Domains, and Gene Structure of CcCNGC Genes

Based on the genomic sequences and annotation information in the citrus genome database, gene structure visualization analysis of *CcCNGC* genes was performed, including intron/exon and untranslated region (UTR) information, using the GSDS 2.0 online website (http://gsds.gao-lab.org/). Conserved domain analysis of citrus CNGC protein sequences was performed using the CDD search tool on the NCBI website. Finally, conserved motif analysis of citrus CNGC protein sequences was conducted using the MEME online tool (https://meme-suite.org/meme/tools/meme, accessed on 20 March 2024).

### 4.5. Chromosomal Location and Collinearity Analysis of CcCNGC Genes

Gene density analysis was performed on the citrus genome using TBtools software version 2.0, and the chromosome position information of *CcCNGC* genes was plotted for visualization analysis [60]. Subsequently, MCScanX version 1.0.0 was used for collinearity analysis, and the results were plotted for collinearity analysis.

### 4.6. Cis-Acting Element Analysis of CcCNGC Gene Family

The sequence of the upstream 2000 bp region of the start codon of *CcCNGC* genes was used as the promoter, and the PlantCARE website (http://bioinformatics.psb.ugent.be/webtools/plantcare/html/, accessed on 25 March 2024) was used to predict cis-acting elements in the sequence. The predicted results were visualized using TBtools software, and the number of elements in each promoter was plotted as a heat map.

### 4.7. Expression Analysis of CcCNGC Genes in Different Tissues

RNA was extracted from roots, stems, leaves, flowers, fruit peel, and fruit pulp of *C. clementina* using the TAKARA Mini BEST Plant RNA Extraction Kit (Thermo Fisher Scientific, Waltham, MA, USA). RNA quality was analyzed by agarose gel (1.5%) electrophoresis and spectrophotometer measurements (NanoDrop 2000, Thermo Fisher Scientific, Waltham, MA, USA). The RNA was then reverse transcribed into cDNA using PrimeScript TMRT Reagent Kit with gDNA Eraser (TaKaRa Dalian, China). Quantitative primers were designed for the citrus CNGC family genes using Primer Premier 5 software version 5.0. The sequence of primers used in this study is listed in Supplementary Table S1. *CsActin* was used as an internal reference gene for expression analysis [61]. The quantitative real-time PCR (qPCR) for *CcCNGCs* was performed on a QuantStudio 5 real-time PCR system (Thermo Fisher Scientific, Waltham, MA, USA) using the SYBR Green mix (Bio-rad, Hercules, CA, USA), with 3 replicates. A PCR mixture totaling 20 μL was prepared, containing cDNA, SYBR Green PCR mix from Applied Biosystems, and specific primer pairs for either the target or reference gene. The thermocycler was programmed as follows: pre-incubation at 95 °C for 10 min followed by 40 cycles of denaturation at 95 °C for 5 s and annealing at 60 °C for 20 s. This was followed by the melt curve stage at 95 °C for 15 s, 60 °C for 1 min, and 95 °C for 15 s. The raw qPCR data were acquired by the QuantStudio^TM^ Design & Analysis Software v1.4.3. The relative gene expression levels of *CcCNGCs* were calculated using the 2^−ΔΔCt^ method to analyze their expression levels in different citrus tissues [62].

### 4.8. Analysis of Expression Levels of CcCNGC Genes Under Different Treatments

Three-month-old *C. clementina* tissue culture seedlings were taken out, wounds were made on the stems, and they were inoculated with *P. nicotianae*, then placed in a sealed box lined with sterilized water-soaked filter paper. Sampling was conducted at time intervals of 0 h, 3 h, 6 h, 12 h, 24 h and 48 h. The samples were immediately frozen in liquid nitrogen and stored in a −80 °C freezer.

Using 2-year-old *C. clementina* as materials, 100 μm GA3, 2 μm SA, 100 μm Indole-3-acetic acid (IAA), 200 μm ABA, and 20 μm methyl jasmonate were sprayed on the *C. clementina*. Leaves were collected at 0 h, 3 h, 6 h, 12 h and 24 h after treatment, quickly frozen in liquid nitrogen, and stored at −80 °C.

The 2-year-old *C. clementina* was subjected to low-temperature stress treatment at 4 °C. Leaf samples were collected at 0 h, 3 h, 6 h, 12 h, 24 h, and 48 h intervals, rapidly frozen in liquid nitrogen, and stored at −80 °C for further analysis.

After 24 h of dark treatment, the 2-year-old *C. clementina* was immediately subjected to light treatment. Samples were taken at 0 h, 3 h, 6 h, 12 h, and 24 h after dark or light treatment, quickly frozen in liquid nitrogen, and stored at −80 °C. Three biological replicates were used for all treatments in this study.

After collecting all the processed samples, the cDNA of the samples extracted as described above was used as a template to detect the relative expression levels of *CcCNGCs* through qRT-PCR by using the 2^−ΔΔCt^ method [62].

### 4.9. Overexpression of CcCNGC Genes in Response to Phytophthora Infection

Based on the results of Experiment 2.8, three family members, namely *CcCNGC21*, *CcCNGC24*, and *CcCNGC27*, exhibited significant responses to *P. nicotianae* stress. The full-length sequences of these genes were amplified using cDNA from *C. clementina* leaves as a template and then cloned into the Super1300 vector by the Seamless Cloning and Assembly Kit (Accurate Biology, Changsha, China). The constructs were then transformed into *Agrobacterium* strain GV3101 using the freeze/thaw method and transient transformation into tobacco leaves. Twenty-four hours later, *P. nicotianae* blocks that had been cultured for 4–5 days were inoculated onto the transiently injected tobacco leaves. Phenotypes were observed, and changes in lesion size were quantified using ImageJ software version 1.8.0.

## 5. Conclusions

In this study, a total of 33 CcCNGC proteins were identified and analyzed from the genome of *C. clementina*, with a focus on their potential roles in citrus defense mechanisms and stress responses. Comprehensive bioinformatics analysis revealed varying levels of structural and functional differentiation among the members of this gene family, consequently resulting in distinct roles played by individual family members in citrus physiology.

## Figures and Tables

**Table 1:** Analysis of physicochemical properties of CcCNGCs.

Protein Name	MW	pI	Protein Length	Instability Index	Aliphatic Index	GRAVY	Localization Predicted
CcCNGC1	95109.31	7.33	834	37.15	31.53	0.755	Plasma Membrane
CcCNGC2	93371.57	6.43	817	40.23	29.82	0.656	Plasma Membrane
CcCNGC3	84175.39	7.04	732	40.36	29.37	0.664	Plasma Membrane
CcCNGC4	81613.47	9.14	710	41.92	27	0.638	Plasma Membrane
CcCNGC5	81613.47	9.14	710	41.92	27	0.638	Plasma Membrane
CcCNGC6	70096.16	8.53	608	43.35	30.54	0.729	Plasma Membrane
CcCNGC7	57963.78	8.79	502	43.65	27.16	0.666	Plasma Membrane
CcCNGC8	57963.78	8.79	502	43.65	27.16	0.666	Plasma Membrane
CcCNGC9	54232.64	8.54	472	44.46	29.03	0.7	Plasma Membrane
CcCNGC10	54232.64	8.54	472	44.46	29.03	0.7	Plasma Membrane
CcCNGC11	51910.01	8.54	445	44.99	32.06	0.756	Plasma Membrane
CcCNGC12	66225.86	8.38	573	44.7	26.88	0.689	Plasma Membrane
CcCNGC13	98268.66	9.24	857	45.57	26.84	0.685	Plasma Membrane
CcCNGC14	59415.25	9.31	507	45.04	30.83	0.712	Plasma Membrane
CcCNGC15	52188.96	9.00	453	39.51	30.02	0.695	NONE
CcCNGC16	99703.54	7.92	888	45.3	27.59	0.757	Plasma Membrane
CcCNGC17	99881.44	6.79	886	44.96	27.24	0.707	Plasma Membrane
CcCNGC18	84197.86	9.00	736	42.66	26.99	0.665	Plasma Membrane
CcCNGC19	83201.18	9.38	723	42.24	26.92	0.677	Plasma Membrane
CcCNGC20	80863.76	8.80	696	40.87	27.44	0.719	Plasma Membrane
CcCNGC21	81978.69	9.51	714	42.44	24.51	0.743	Plasma Membrane
CcCNGC22	81754.89	9.33	711	43.06	27.19	0.717	Plasma Membrane
CcCNGC23	76781.88	9.47	668	39.74	25.35	0.685	Plasma Membrane
CcCNGC24	89800.61	6.53	785	45.84	31.04	0.805	Plasma Membrane
CcCNGC25	73690.31	9.41	637	45.59	28.99	0.721	Plasma Membrane
CcCNGC26	83295.60	8.24	732	45.73	25	0.723	Plasma Membrane
CcCNGC27	83314.54	9.17	732	37.06	27.34	0.671	Plasma Membrane
CcCNGC28	79175.28	7.87	691	48.11	27.21	0.738	Plasma Membrane
CcCNGC29	94303.40	6.42	822	40.07	29.68	0.706	Plasma Membrane
CcCNGC30	83937.67	9.23	730	35.55	27.12	0.616	Plasma Membrane
CcCNGC31	84393.41	9.26	734	39.98	26.66	0.64	Plasma Membrane
CcCNGC32	80206.90	9.30	698	40.41	27.98	0.72	Plasma Membrane
CcCNGC33	75034.25	9.34	650	45.23	28.82	0.739	Plasma Membrane

## Data Availability

The original contributions presented in this study are included in the article and Supplementary Materials. Further inquiries can be directed to the corresponding author.

## Supplementary Materials

The following supporting information can be downloaded at: https://www.mdpi.com/article/10.3390/ijms26030960/s1.

## Abbreviations

*C. clementina*	*Citrus clementina*
*C. sinensis*	*Citrus sinensis*
*P. trifoliata*	*Poncirus trifoliata*
*P. infestans*	*Phytophthora infestans*
*A. thaliana*	*Arabidopsis thaliana*
*N. tabacum*	*Nicotiana tabacum*
IAA	Indole-3-acetic acid
MeJA	Methyl Jasmonate
SA	Salicylic acid
ABA	Abscisic acid
GA	Gibberellins
