# Supplementary material for: Analysis of CNGC Family Members in Citrus clementina (Hort. ex Tan.) by a Genome-Wide Approach
Source: Int J Mol Sci. 2025 Jan 23;26(3):960. doi: 10.3390/ijms26030960 (PMC11817026; doi:10.3390/ijms26030960)
Supplement: Supplementary file 1 [file ijms-26-00960-s001.zip › ijms-3380197-supplementary.pdf]

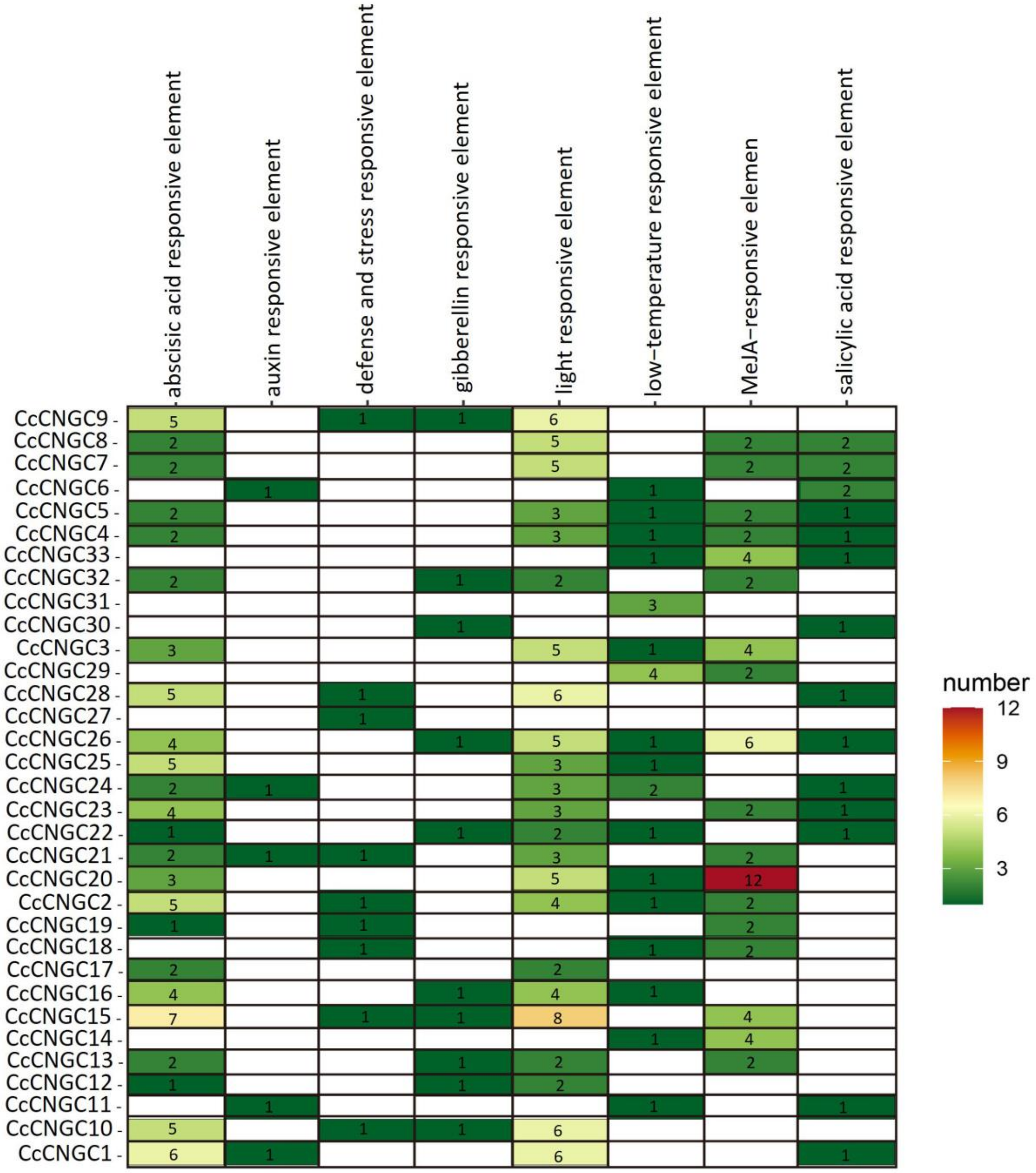


**Figure S1.** Cis-acting element number heat map of *CcCNGC* genes.

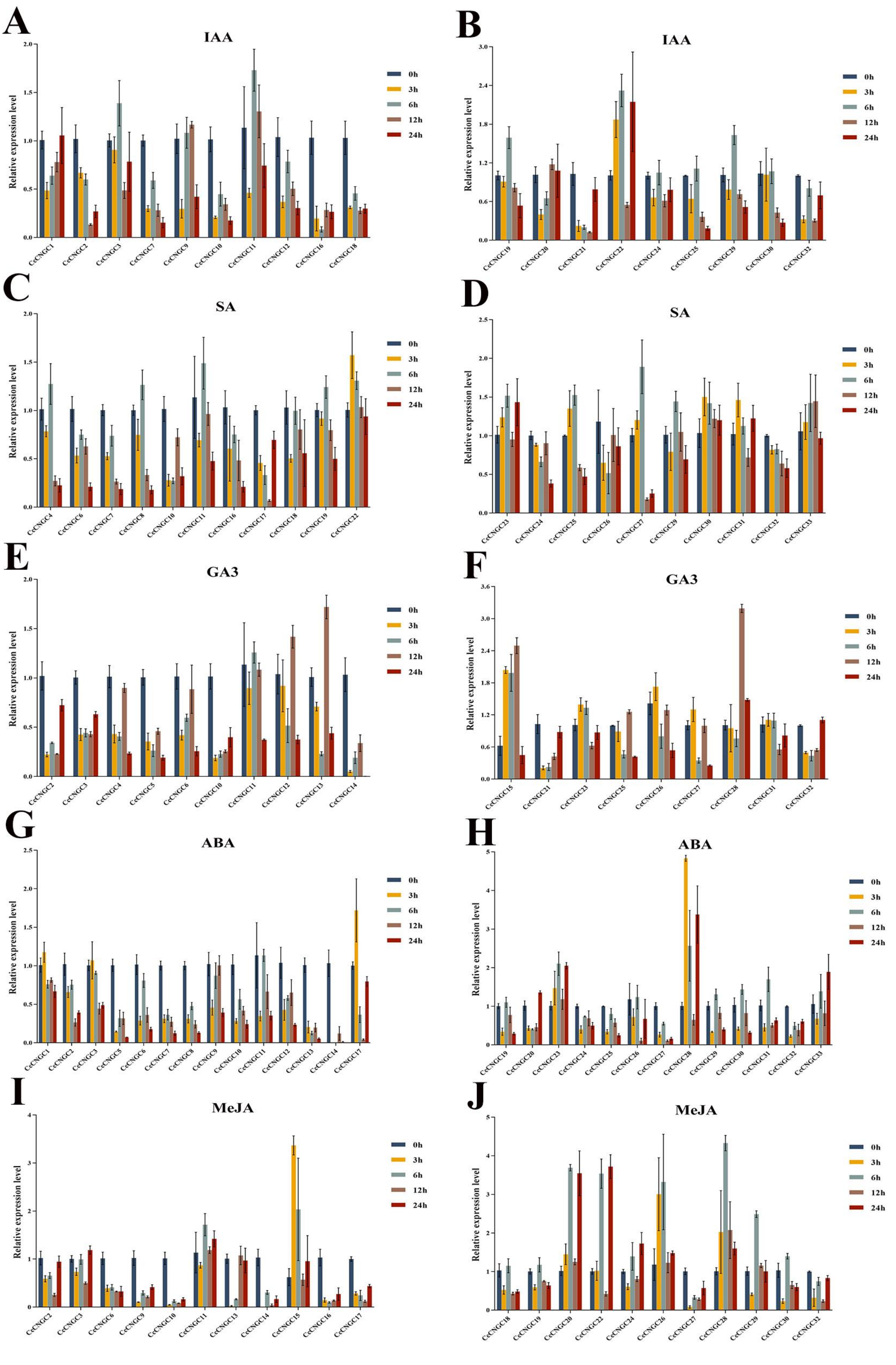


**Figure S2.** The expression of *CcCNGC* genes under IAA, SA, GA3, ABA and MeJA treatment.

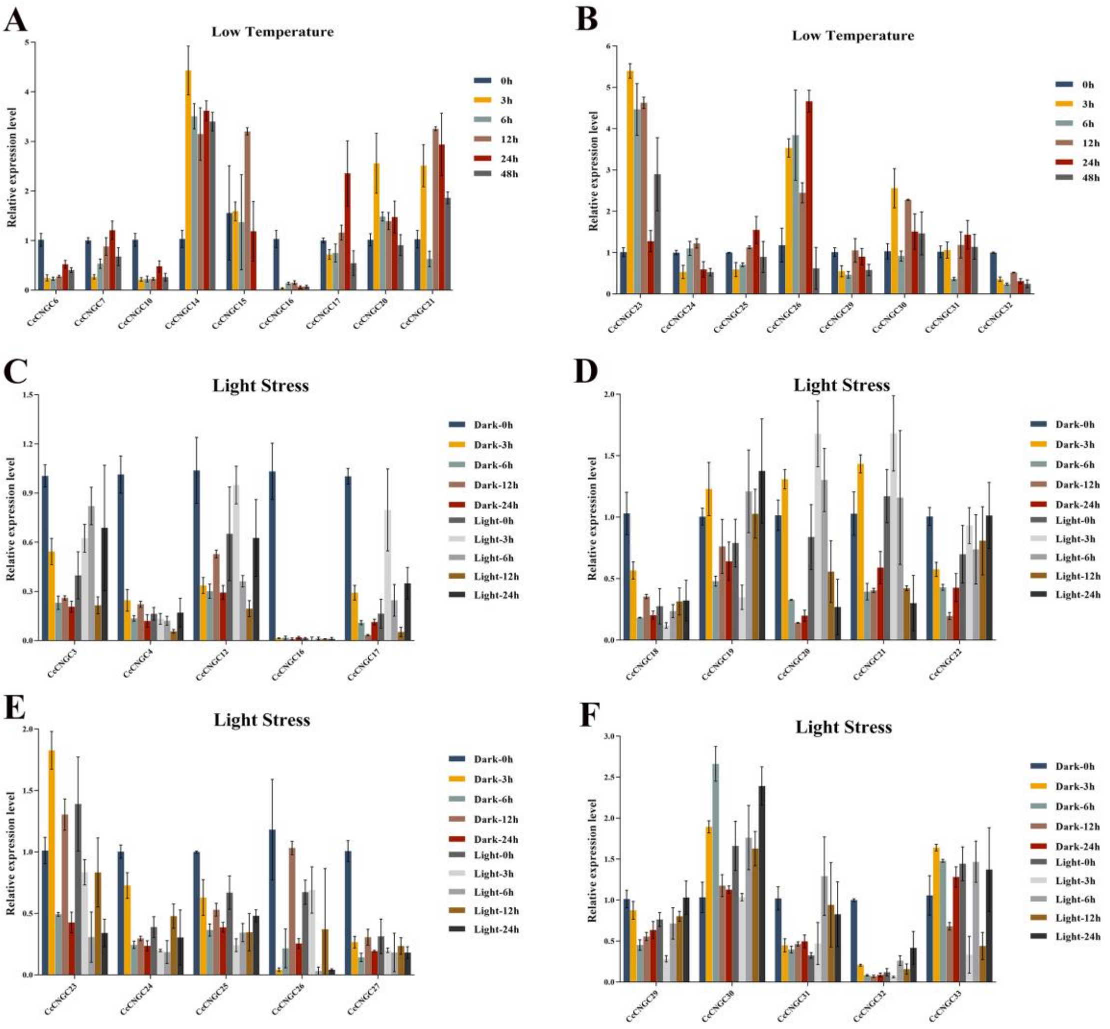


**Figure S3.** The expression of *CcCNGC* genes under low temperature and light stress.

**Supplemental Table S1.** The Primers of this work.

| Primer | sequence |
|---|---|
| *CcCNGC1*-qF | CACCGCCATCTCCGATCCCTATA |
| *CcCNGC1*-qR | CATCTGACCCGTTCATCACCAAA |
| *CcCNGC2*-qF | CATGGTTCTTGGAGCTTACTTGA |
| *CcCNGC2*-qR | ATCTTAGCACGAATGGAGACAGG |
| *CcCNGC3*-qF | GCTGGAGCTGATCCGAACAAGAC |
| *CcCNGC3*-qR | AAATCTGAATCCCCCCTGGCAACAA |
| *CcCNGC4*-qF | GCTCTAATGGCTGATGACTTG |
| *CcCNGC4*-qR | CATAAATGGTGGCTCCTAAAC |
| *CcCNGC5*-qF | TTGCTCTAATGGCTGATGACTTG |
| *CcCNGC5*-qR | TAAATGGTGGCTCCTAAACTTGG |
| *CcCNGC6*-qF | CAATGAAGACGCGAAATGCGACAA |
| *CcCNGC6*-qR | AGGGCAAACAACAGGAAACCAGAG |
| *CcCNGC7*-qF | GCTCTAATGGCTGATGACTTG |
| *CcCNGC7*-qR | CATAAATGGTGGCTCCTAAAC |
| *CcCNGC8*-qF | GCTCTAATGGCTGATGACTTG |
| *CcCNGC8*-qR | CATAAATGGTGGCTCCTAAAC |
| *CcCNGC9*-qF | GTACGAGAAATAGACCGGTGGCTGC |
| *CcCNGC9*-qR | AGACGACTGGCTTCACAACATCA |
| *CcCNGC10*-qF | CATCATTCTTCCACTCCCACAGG |
| *CcCNGC10*-qR | AACGTCAGATTGGCCCATTTATT |
| *CcCNGC11*-qF | GGAGATGACATTGAAGCCACG |
| *CcCNGC11*-qR | TCTGAGTTCATTGGGAAGATTATTG |
| *CcCNGC12*-qF | TAACTATAAAGTCGGAGAATGTGAG |
| *CcCNGC12*-qR | TAAGGTCCTTGGGAAGATTGAGC |
| *CcCNGC13*-qF | TTTCAAAGCGGTTTCTATCTCAG |
| *CcCNGC13*-qR | GGTTACAGCCCTCAAGTTATCAG |
| *CcCNGC14*-qF | AGTAATAGTCGTTGTCGTAGAGAGG |
| *CcCNGC14*-qR | GCGATTGCGATAGTGTTCCAAGTCT |
| *CcCNGC15*-qF | AAACATCCCTTCAGCAGATTCGT |
| *CcCNGC15*-qR | TGCATACAATCGCTTAACCCTCA |
| *CcCNGC16*-qF | TACAGTTACATTCGGGTTCGCTAC |
| *CcCNGC16*-qR | TTGTTTGAAGTCTCCCAGGGTTT |
| *CcCNGC17*-qF | AAGAAGCAACCGTAGAGTCAAGC |
| *CcCNGC17*-qR | GTAAGCCACAAAGAAAGTGAGAATG |
| *CcCNGC18*-qF | TTGAAAGTGTAACGACAGATGGTGGAA |
| *CcCNGC18*-qR | GCCTGAACTGACTAGCGACGAAT |
| *CcCNGC19*-qF | CTTGTTCGACGTGTCCCATTCTTCT |
| *CcCNGC19*-qR | CCAGTCCGACCTCCGTTAGTTGT |
| *CcCNGC20*-qF | CTGCCTGTTGCGGCGTAT |
| *CcCNGC20*-qR | TCTTAGGCATTTTGCTGACCTCTGA |
| *CcCNGC21*-qF | GGCGAAAACAACAACGACG |

| | |
|---|---|
| *CcCNGC21*-qR | GAATGGGGACGAGGGAGG |
| *CcCNGC22*-qF | GTCATCCTCCCCTCATCCAC |
| *CcCNGC22*-qR | ATGCGGCCCACGTTCTC |
| *CcCNGC23*-qF | CTGGCACTGATTGTGACGGT |
| *CcCNGC23*-qR | TGCCTGAGGAATCGGTAGTATG |
| *CcCNGC24*-qF | GTGCCAGACACCAAGACTATCC |
| *CcCNGC24*-qR | GCATTCAGTTCTGTTTGACCATCTT |
| *CcCNGC25*-qF | GCTCTAATGGCAGATGACTTGAA |
| *CcCNGC25*-qR | CGCATAAATGGTGGCTCCT |
| *CcCNGC26*-qF | ATGTATCCTTGGTTCGGTCTTGTTT |
| *CcCNGC26*-qR | CGACGGAGGTCCACAGGTAG |
| *CcCNGC27*-qF | GGCGTGATGTATGTCGGAAGT |
| *CcCNGC27*-qR | ACCCCACCAAAAGCAGTAAAA |
| *CcCNGC28*-qF | GATTTCTTGGTCGGGGTCAG |
| *CcCNGC28*-qR | GCCAAGCATTGCGTTCAG |
| *CcCNGC29*-qF | ATGATTTGAACGAAAGCCTGAG |
| *CcCNGC29*-qR | AATTTTGTCCATGCCCGATAC |
| *CcCNGC30*-qF | CTATTCGCAGCAATGGAGGA |
| *CcCNGC30*-qR | ACGGAGCATCACTAAAATCAACA |
| *CcCNGC31*-qF | AGGAAGAAAGCAACTGAACAACG |
| *CcCNGC31*-qR | CATTAGCGGCAAAGCGAGA |
| *CcCNGC32*-qF | AGTTGAGGCTTTTGCTTTACGA |
| *CcCNGC32*-qR | TGAAGGAGGCTGGGGAGA |
| *CcCNGC33*-qF | TGATTTCTGTGGCGAGGAGTT |
| *CcCNGC33*-qR | CTTGTGCCTAAGTTGCTTGCTAT |
| *CcCNGC21*-OE-F | CAAATCGACTCTAGAAAGCTT |
| | ATGCCCTCTCACCCCAACTTCCCCT |
| *CcCNGC21*-OE-R | CACTAGTATTTAAATGTCGAC |
| | TTCAAGGTGATCATGTGGTCTA |
| *CcCNGC24*-OE-F | CAAATCGACTCTAGAAAGCTT |
| | ATGTCTTTTTCTTGTGCCAAAAACT |
| *CcCNGC24*-OE-R | CACTAGTATTTAAATGTCGAC |
| | GCTCACATCTTTACCCATGGTT |
| *CcCNGC27*-OE-F | CAAATCGACTCTAGAAAGCTT |
| | ATGAATACCGAGAGAGTTAAATTTG |
| *CcCNGC27*-OE-R | CACTAGTATTTAAATGTCGAC |
| | ATCTTTGGCAGTGAAGTCAGGC |
| ACTIN-F | ATCTGCTGGAAGGTGCTGAG |
| ACTIN-R | CCAAGCAGCATGAAGATCAA |
